# An Ontology Systems Approach on Human Brain Expression and Metaproteomics

**DOI:** 10.3389/fmicb.2018.00406

**Published:** 2018-03-08

**Authors:** Adolfo Flores Saiffe Farías, Adriana P. Mendizabal, J. Alejandro Morales

**Affiliations:** ^1^Computer Sciences Department, University of Guadalara, Guadalajara, Mexico; ^2^Pharmacobiology Department, University of Guadalajara, Guadalajara, Mexico

**Keywords:** gene ontology, microbiota-gut-brain axis, brain structures, brain physiology, metaproteome, gene silencing, ion channel, Parkinson disease

## Abstract

Research in the last decade has shown growing evidence of the gut microbiota influence on brain physiology. While many mechanisms of this influence have been proposed in animal models, most studies in humans are the result of a pathology–dysbiosis association and very few have related the presence of certain taxa with brain substructures or molecular pathways. In this paper, we associated the functional ontologies in the differential expression of brain substructures from the Allen Brain Atlas database, with those of the metaproteome from the Human Microbiome Project. Our results showed several coherent clustered ontologies where many taxa could influence brain expression and physiology. A detailed analysis of psychobiotics showed specific slim ontologies functionally associated with substructures in the basal ganglia and cerebellar cortex. Some of the most relevant slim ontology groups are related to *Ion transport, Membrane potential, Synapse, DNA and RNA metabolism*, and *Antigen processing*, while the most relevant neuropathology found was Parkinson disease. In some of these cases, new hypothetical gut microbiota-brain interaction pathways are proposed.

## 1. Introduction

Recently, strong evidence has related the gut microbiota with almost all of the host physiology, including the brain, behavior and cognition. Experiments with both, manipulation of the gut microbiota in stress and germ–free animals, have disclosed a bidirectional communication system between the gut microbiota and the central nervous system: the microbiota-gut-brain axis (MGBa) (Dinan and Cryan, 2016, 2017). The gut microbiome handles hundreds of thousands of different proteins and metabolites, some of which are neuroactive components, and thus can communicate with the host brain, via the peripheral nervous system or through the Blood-Brain Barrier, affecting various molecular pathways (Wall et al., 2014; Dinan and Cryan, 2017). Growing evidence in humans strongly suggests that these microbial neuroactive components not only play an essential role in regulating synaptic circuit activation and neurodevelopment, but they can influence the host's emotions, behavior and cognition (Borre et al., 2014; Rea et al., 2016; Sarkar et al., 2016; Foster et al., 2017). These studies have also revealed that dysbioses, the gut micorbiota alterations or insults, promotes brain-associated diseases and disorders like Parkinson's disease (PD), anxiety and many others (Dinan and Cryan, 2017; Wiley et al., 2017).

Most of the human dysbiosis-associated neurological conditions are the result of statistical approaches using behavioral or cognitive variables, this is due to the complications of performing molecular studies in viable human brains. Although a few communication mechanisms have been suggested within the MGBa (e.g., the metabolism of tryptophan and gastrointestinal hormones microbiota dependent, and the interaction of microbiota dependent signaling molecules to the vagus nerve Wiley et al., 2017), many of them are still unknown. Thus, the complex mechanisms underlying cognition and behavior remain largely uncharacterized.

Here we hypothesize that gut taxa could be coherently associated with regions of the human brain by using functional annotations to provide a conceptual framework of putative influence mechanisms of the microbiota with the brain. We designed an *in silico* pipeline based on metaproteome (the set of microbiotal proteins) and brain expression data processed by sequence alignment tools and Gene Ontology (GO) functional groups, or slims. To our knowledge, this is the first study where whole metaproteome is functionally associated to differential expression patterns in brain regions using a blind systems approach.

## 2. Results

### 2.1. Data curation

We obtained 92 non-redundant metaproteome datasets: one per taxon at the genus level. All protein sequences from each dataset were PSI-blasted against the Human Protein Reference Sequences (RefSeq-prot). The resulting non-redundant Blast hits in each taxon were enriched with functional gene ontologies (GOs). Statistically non-significant GOs were filtered-out. Table S1 contains the number of metaproteins, their hits to the RefSeq-prot and their ontologies found per taxon.

**Figure 1:**
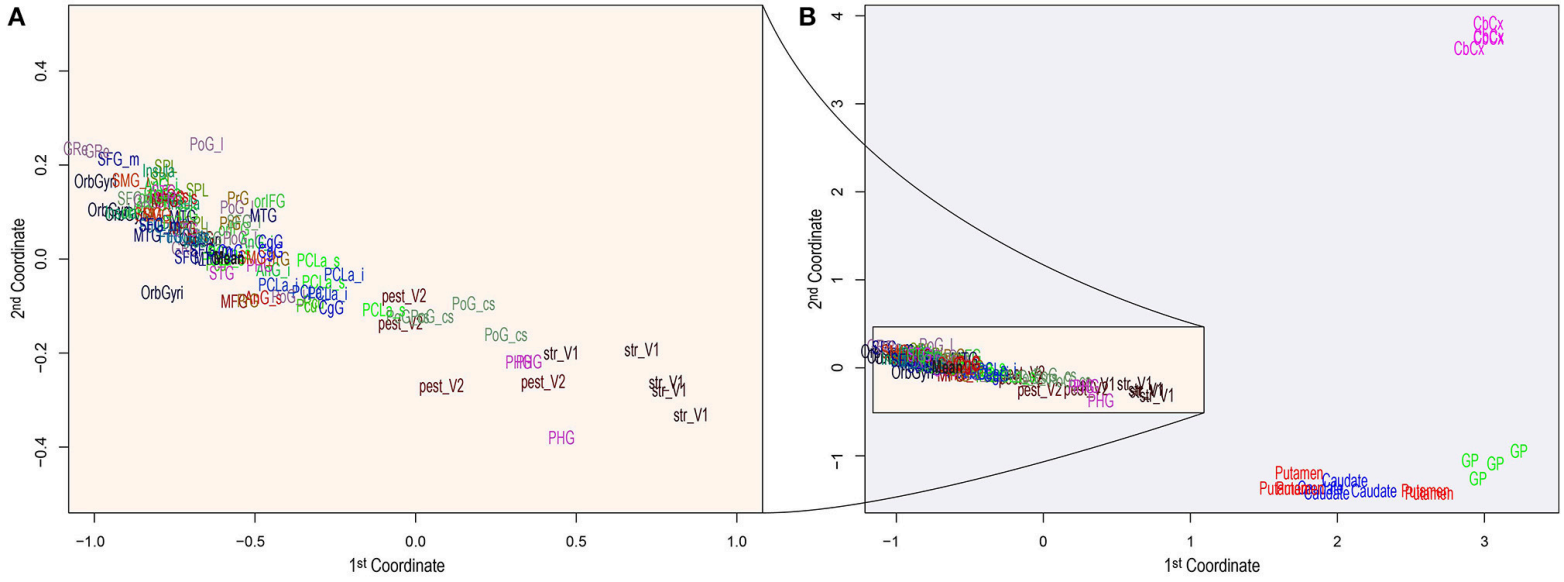
Principal Component Analysis of brain distances obtained using the 500 most informative genes among substructures. The two principal components or coordinates are plotted on the x– and y–axis. The entire component space **(A)** zooms the region of higher density, while **(B)** depicts all substructures, showing the clear separation of a few substructures from the rest. Acronym-to-name relations are presented in Table 1.

The RNA-seq data from the Allen Brain Atlas, containing 22,318 genes, was filtered (detailed in the section 5) and resulted in 16,242 genes (72.78%). Figure 1A shows the leading log2–fold–change Euclidean distances between samples by substructure abbreviation (see Table 1), where some substructures are separated from the rest by their differential expression patterns Figure 1B. Table S2 contains the log2 difference between the mean counts per million (CPM) from all samples with the CPM of each sample, the *F*-value, *p*-value and Bonferroni's false discovery rate of testing for differential expression between samples. We selected the genes differentially expressed, according to the mean expression from all samples. Expressed genes by brain substructure were enriched with functional GOs, and only the statistically significant were preserved. Table S3 contains both, the number of differentially expressed genes and GOs found in enrichment per brain substructure.

**Table 1:** Brain substructure name and their abbreviations.

**Abbreviation**	**Substructure**	**Abbreviation**	**Substructure**
AnG_i	Angular gyrus inferior	AnG_s	Angular gyrus superior
Caudate	Body of the caudate nucleus	CbCx	Cerebellar cortex
CgG	Cingulate gyrus	FuG_i	Fusiform gyrus lateral
GP	Globus pallidus	GRe	Gyrus rectus
Insula	Long insular gyri	ITG	Inferior temporal gyrus
MFG	Middle frontal gyrus	MTG	Middle temporal gyrus
OrbGyri	Lateral orbital gyrus	orIFG	Inferior frontal gyrus orbital part
PCLa_i	Paracentral lobule anterior inferior	PCLa_s	Paracentral lobule anterior superior
Pcu	Precuneus	pest_V2	Cuneus peristriate
PHG	Parahippocampal gyrus	PoG_cs	Post-central gyrus central sulcus
PoG_l	Post-central gyrus_lateral	PrG	Pre-central gyrus
Putame	Putamen	SFG_l	Superior_frontal gyrus lateral
SFG_m	Superior frontal gyrus medial	SMG_i	Supramarginal gyrus inferior
SPL	Superior parietal lobule	STG	Superior temporal gyrus
str_V1	Lingual gyrus striate

**Figure 2:**
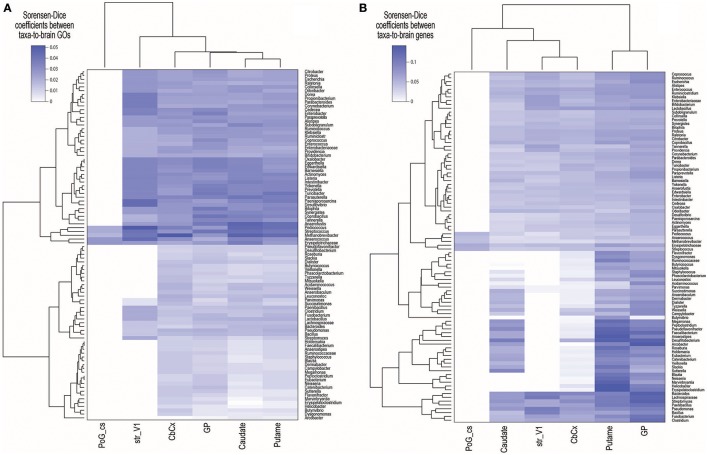
Sorensen–Dice (SD) coefficients heatmap of **(A)** common Gene Ontologies (GOs) and **(B)** genes across brain substructures and taxa. SD coefficient rage values are zero to one, where zero means completely dissimilar and one means identical sets. Acronym–to–name relations are presented in Table 1.

We found 4,599 taxa–to–brain substructure (T2BS) common GOs (see Table S4). From these 108 were unique GOs, 92 taxa and six brain substructures. Figures 2A,B show the Sorensen–Dice coefficient of the GOs and genes found in each taxon vs. each substructure respectively. To test if the number of proteins found by Blast and subsequently the number of matching GOs are biased by the number of metaproteins per taxon, we performed a Pearson's correlation between the latter. The resulting value of −0.55 indicates that there is no direct correlation between the number of metaproteins per taxon and the number of GOs (see Figure S1).

### 2.2. GO slims

We grouped the 108 unique GOs found, by calculating their semantic similarity (see section 5) among all of them. We applied hierarchical clustering (see Figure S2) to the distances and manually grouped them into coherent clusters with similar function, resulting in a total of 14 slims (see Tables S4, S5). The ontological maps for each slim can be found at Figure S3.

**Figure 3:**
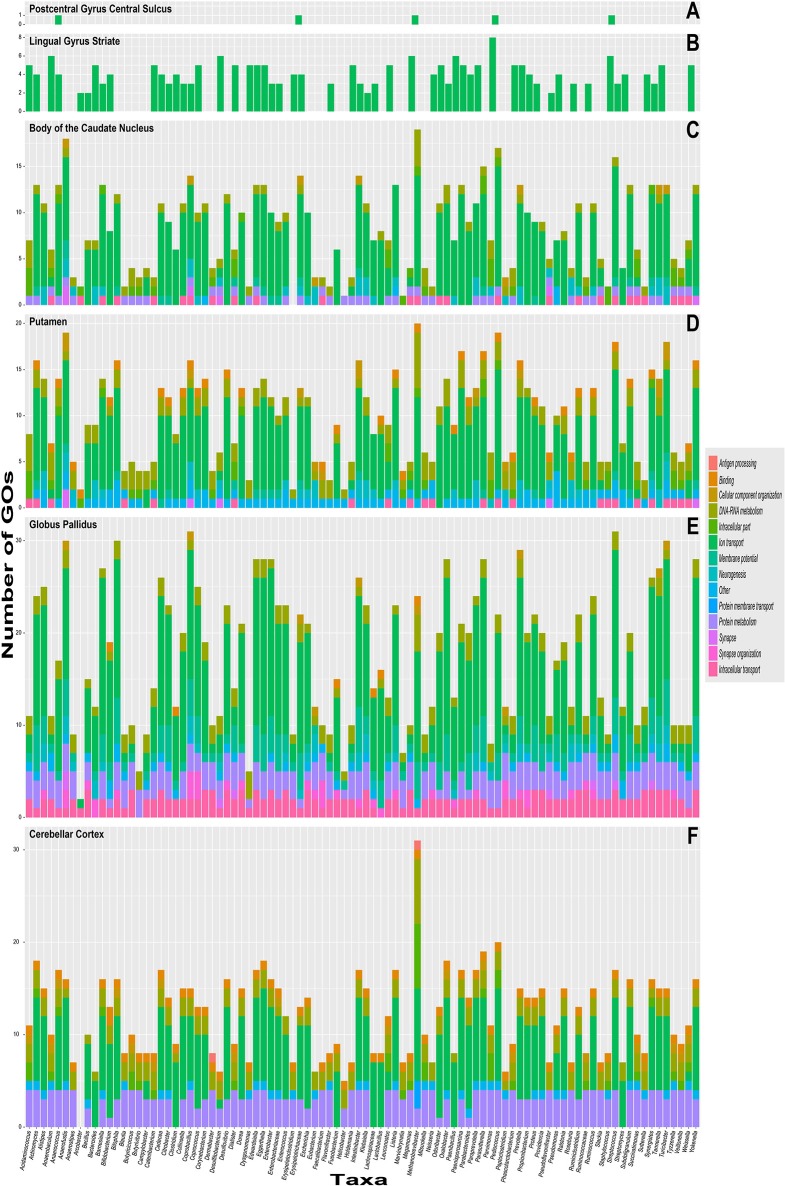
Stacked bar graphs with the quantity of common taxa–to–brain substruture Gene ontology labels on the y–axis and color-coded slims. The x–axis has each of the 92 different genre analyzed. Each graph represent brain substructures **(A)** Postcentral gyrus central sulcus, **(B)** Lingual gyrus striate, **(C)** Body of the caudate nucleus, **(D)** Putamen, **(E)** Globus pallidus and **(F)** Cerebellar cortex.

Figure 3 shows the number of common GOs between taxon and brain substructure, colored by slims. We can observe that the most frequent slim is *Ion transport*, followed by *Protein metabolism* and *DNA and RNA metabolism*. Also, the Globus pallidus is the substructure where more associations were found, followed by the Cerebellar cortex. Table 2 shows the GOs, taxa, and brain substructures count per slim.

**Table 2:** Substructures and counts of GOs and taxa by slim.

**Slim**	**GOs**	**Taxa**	**Substructure**
Ion transport	30	85	GP, Caudate, CbCx, Putame, str_V1, PoG_cs
Membrane potential	6	86	Caudate, GP, Putame
Protein membrane transport	3	1	CbCx
Synapse	6	9	Caudate, GP, Putame
Synapse organization	3	27	GP
Transport (others)	7	89	Caudate, GP, Putame
Antigen processing	2	2	CbCx
Binding	4	85	CbCx, Putame, GP
Cellular component organization	4	31	CbCx, Caudate, GP, Putame
DNA and RNA metabolism	15	90	CbCx, Caudate, GP, Putame
Intracellular part	10	28	Caudate, CbCx, Putame, str_V1
Neurogenesis	2	28	Caudate, GP, Putame
Other	10	89	CbCx, Caudate, GP, Putame
Protein metabolism	6	84	CbCx, Caudate, GP

## 3. Discussion

The comorbidity between dysbiosis and cognitive or behavioral impairment has sparked a race to understand the mechanisms of these associations. Since then, researchers have glimpsed the influence of microbiota in behavior and cognition, and several interaction pathways have been proposed via the Blood Brain Barrier or the vagus nerve, involving neuropeptides (Holzer and Farzi, 2014), inflammatory molecular signaling (Rook et al., 2014), hormones (Rehfeld, 2014), microRNAs (miRNAs) (Hoban et al., 2017a), among others (Wall et al., 2014). In our study, the correlation between the brain proteins and the metaproteome into functional ontologies supports these observations.

Advances in sequencing technology have paved the way for the creation of reference databases in many fields of research. The Human Microbiome Project has consistently sequenced the microbiota from different body parts and created the Reference Genome Database body part-specific. On the other side, the Allen Brain Atlas organization has performed RNA-seq (quadruplicate at least) of 29 different brain substructures in two post-mortem subjects. Despite this sampling being biased (due to post-mortem) and underpowered, it enabled us to perform this work as a “test drive.” Our aim was not to prove a direct link between gene expression levels in the brain and the presence of specific taxa but to strengthen the evidence of known MGBa mechanisms as well as to uncover putative new avenues of research in the axis.

The analysis pipeline, being a data–driven approach, is prone to false positives. Thus we have used multiple-comparisons correction methods, to increase the proportion of true positives (at the expense of false negatives, though). From the 29 substructures, only six of them were found to have common GO annotations with those associated with microbiota. These six substructures (Cerebellar Cortex, Globus pallidus, Putamen, Body of the caudate nucleus, Lingual gyrus striate and Postcentral gyrus central sulcus) appear distant from the rest (Figure 1), which means that they have different and broader expression patterns than most of the substructures and will have more significant enriched GOs (see Table S3).

The tremendous complexity of the human brain has limited the approaches to the MGBa. Most of such studies measure behavioral responses involving different types of memory or stress, while only a few associate cognition or behavior with specific brain regions, circuits, pathways, and taxa. Assuming that cognitive function is associated with structural micro-connectivity and specific gene expression patterns (across cell types) regulating input and output signals, this work is based on the paradigm that cognition is the result of communication patterns that emerge from the interaction of specialized brain substructures connected in certain circuitry across several molecular pathways. Our methodology is designed to find common T2BS functional annotations, based on differential expression of brain structures and the taxa metaproteome, assuming that portions of the latter are expressed under certain conditions.

Given that we cannot assume that homology of a metaprotein with a human brain gene is only associated due to its similarity, we have turned to a differential functional approach. Gene enrichment method is used here to find groups of genes overrepresented with a similar function. Such gene–function association allows us to perform more robust T2BS associations.

The resulting common GOs clustered naturally according to their semantic distances in the ontology map. With these, we performed *a posteriori* design of GO slims that coherently clustered similar GO annotations. These slims enabled us to analyze and discuss our results by functionally coherent groups.

### 3.1. Pyschobiotic and slim selection

Psychobiotics are microorganisms that have a positive influence on the mental health when ingested in adequate amounts (Dinan and Cryan, 2017). Several bacteria have been proposed as such, and we have selected those genera with consistent evidence of mental health influence or neurotransmitter-producing capabilities.

There is evidence of *Actinomyces, Bifidobacterium*, and *Faecalibacterium* having positive effects on anxiety and/or depression (Messaoudi et al., 2011; Jiang et al., 2015; Kelly et al., 2016; Zheng et al., 2016) and *Bacteroides, Prevotella*, and *Lactobacillus* in autism spectrum disorder. *Bifidobacterium* ameliorates the hypothalamic-pituitary-adrenal system under stress in germ-free mice (Sudo et al., 2004). Tillisch *et al*. tested a healthy women population found that increased abundance of *Prevotella* showed differential response to negatively valenced images and greater white matter connectivity in limbic–cortical–striatal–pallidal–thalamic circuitry, and smaller hippocampal volume in comparison with the *Bacteroides*-high group. The *Prevotella*-high group was also found to have higher connectivity in the temporal lobe (Tillisch et al., 2017). Sheperjans et al. conducted a case–control study of 72 subjects with Parkinson's disease and found reduced *Prevotella* in the feces of case–subjects, and the abundance of *Enterobacteriaceae* correlated with postural instability and gait difficulty (Scheperjans et al., 2015). We have also considered as psychobiotics those microorganisms able to produce neurotransmitters like *Bacillus, Bifidobacterium, Escherichia, Enterococcus, Lactobacillus, Staphilococcus*, and *Streptococcus* (Horiuchi et al., 2003; Bravo et al., 2011; Barrett et al., 2012; Lyte, 2014; Wall et al., 2014; Desbonnet et al., 2015; Dinan and Cryan, 2017). For example, Bravo et al., in 2011 studied mice with a *Lactobacillus* treatment and found altered expression of GABA receptors, vagous nerve-dependent, in cortical regions, hippocampus, amygdala and *locus coerulus* and reduced anxiety and depression–related behavior (Bravo et al., 2011). Based on the evidence here discussed, we have tagged the mentioned bacteria as psychobiotics.

**Figure 4:**
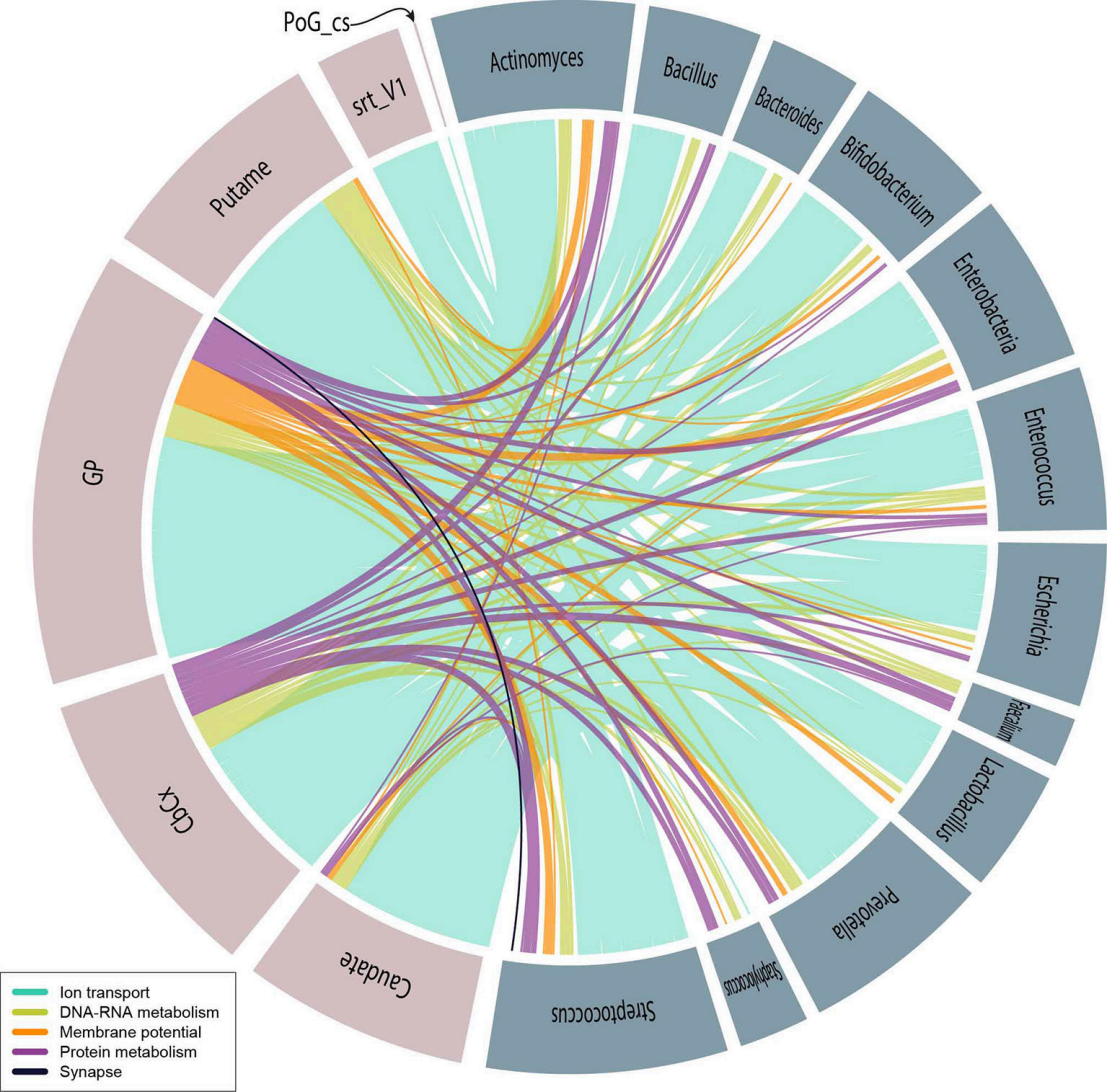
Psychobiotic–brain relationships represented by a colored edge corresponding to the slims of interest as indicated in the caption. Pink–colored circle fractions correspond to brain sub-structure (Abbreviation) and blue–gray–colored circle fractions correspond to the following psychobiotics: *Actinomyces, Bacillus, Bacteroides, Bifidobacterium, Enterobacteriaceae, Enterococcus, Escherichia, Faecalibacterium, Lactobacillus, Prevotella, Staphylococcus*, and *Streptococcus*.

We have selected the slims that could be conceptually directly related to brain activity or the cognition: *Synapse, DNA and RNA metabolism, Protein metabolism, Membrane potential* and *Ion transport*. These slims contained 541 GOs associating T2BS. Figure 4 shows these relationships. Specific discussion of the putative role of psychobiotics (and other microorganisms) within the slims can be found below.

### 3.2. Gut microbiota and brain cells membranes

Behavior and cognition are intrinsically dependent on the communication within the brain, that is electrical impulses and synapses. The flow of electrical impulses is given by the efficient ion movement across the neuron cell membranes through voltage-gated ion channels. Deficiencies in voltage-gated ion channels and synapses have been related to several mental and movement disorders (Baldessarini, 1996; Yogeeswari et al., 2004; Sullivan et al., 2012; Imbrici et al., 2013; Vitaliti et al., 2014; Mourre et al., 2017; Reig-Viader et al., in press; Roeper, 2017). For example, epilepsy (Devergnas et al., 2012; Carecchio and Mencacci, 2017) and PD (Mourre et al., 2017) are associated with the basal ganglia, while ataxia has been observed with ion channel dysfunction in the cerebellum (Waszkielewicz et al., 2013).

On the other hand, gut dysbioses have been previously associated to most of these conditions (Parracho et al., 2005; MacFabe et al., 2011; Rook et al., 2014; Maqsood and Stone, 2016). Sudo et al., and Neufeld et al. reported a decreased expression of subunits of the NMDA receptor (a glutamate and ion channel protein) in both, cortex and hippocampus (Sudo et al., 2004), and in central amygdala in GF-mice (Neufeld et al., 2011). This suggests possible mechanisms of microbiota–mediated synapses and ion channel regulation.

We report a high density of functional associations related to electrical impulses and synapse communication (see Figure S3, slims *Ion transport, Membrane potential, Protein membrane transport, Synapse, Synapse organization*, and *Transport* (*others*)). We have found four ontologies (GO:0005249, GO:0005267, GO:0022843, and GO:0034705) present in more than 50% of the T2BS relations (see Figure S3 and Table S4). Surprisingly these four are part of the *Ion transport* slim, which is related to ion voltage-gated channel activities (see Table S5). Also, more than half of all of the T2BS GO relations are associated by the *Ion transport* slim, especially at the Globus pallidus, Putamen and the Body of the Caudate nucleus (substructures of the basal ganglia), Cerebellum cortex and Striate. Our findings strongly support the hypothesis of the influence of the metaproteome with mental and movement–related neurological disorders by the direct or indirect interaction with ion channels (slim *Ion transport*) and regulation of membrane potential (slim *Membrane potential*).

We have found 89 taxa that putatively influence the basal ganglia at the level of neurotransmitter transport and other chemicals (see the *Transport (others)* ontology map in Figure S3). Also, we have found 27 taxa that could influence the structural organization of synapse at the Globus pallidus (see the *Synapse organization* ontology map in Figure S3). Our results agree with the evidence of microbiota influencing neurotransmitter receptors, like the serotonin receptor 1A (5HT1A) (Sudo et al., 2004) and GABA receptors via the vagus nerve (Bravo et al., 2011), and the altered neurotransmitter levels found in the striatum of GF–mice (Diaz Heijtz et al., 2011).

Other approaches suggest that the gut microbiota can influence synapse function and neurogenesis by influencing the brain-derived neurotrophic factor (BDNF), a key regulator on neurogenesis and synapses (Sudo et al., 2004; Bercik et al., 2011). In this context, we found nine taxa within the *Synapse* slim and 28 taxa within the *Neurogenesis* slim, both associated with the basal ganglia.

By selecting the taxa and slims mentioned in the psychobiotics analysis, we observed that the seven most abundant GOs (all within the *Ion transport* slim), represent 64% of the T2BSs, and 76% of those, are associated with the potassium ion channels (see Figure 4). Also, the Globus Pallidus (34%) was found to share most of mentions followed by the cerebellar cortex, the putamen and the caudate. These results suggest that psychobiotics could influence voltage-gated channels, especially those involved with potassium channels in the basal ganglia. As discussed above, there is evidence of movement disorders associated with basal ganglia and ion channels (Devergnas et al., 2012; Carecchio and Mencacci, 2017; Mourre et al., 2017) and with psychobiotic dysbioses (Scheperjans et al., 2015; Hill-Burns et al., 2017; Li et al., 2017). Also, we have found other GO labels within the slims of *Membrane potential* and *Synapse* which suggests that psychobiotics also play a role in the action potential and synaptic membrane.

### 3.3. Gene expression of the host brain and the influence of gut microbiota

Cognition and behavior disorders are also associated with gene expression processes and their highly complex regulatory mechanisms, which involve miRNAs (a product of splicing) and epigenomic regulatory marks (e.g., DNA methylation, histone modifications, non-coding RNAs). The slim of *DNA and RNA metabolism*, which contains 12.3% of the total T2BS, associates 90 taxa with four brain substructures (see Table 2) through 15 GO terms (GO:0016072, GO:0006399, GO:0006364, GO:0008033, GO:0009451, GO:0004518, GO:0006402, GO:0000375, GO:0000398, GO:0000184, GO:0019083, GO:0071013, GO:0000956, GO:0006353, GO:0016570). Suggesting that the microbiome is capable of regulating host's nucleic acid metabolism via the spliceosome, catabolic processing the RNA, histone modification, RNA modification, rRNA and tRNA processing or nuclease activity based on the GO terms found (see Figure S3 and Table S4).

*Methanobrevibacter*, the most abundant archaea in the human gut, appears in mentions of the spliceosome (GO:0000398, GO:0000375, and GO:0071013) in the Globus pallidus, Putamen, Body of the Caudate nucleus and Cerebellar cortex. The spliceosome is the machinery that regulates transcript RNA splicing, into various RNA functional products, including mRNAs and miRNAs. Hasler et al. found evidence of the microbiota influencing host-gene expression and RNA splicing in host-mucosal cells (Häsler et al., 2016), which suggest the involvement of miRNAs in regulatory mechanisms. These are known to have a role in neuropsychiatric disorders (Alural et al., 2017), anxiety-like behaviors (Hoban et al., 2017b) and movement disorders (Tan et al., 2013). Increased miRNAs have been reported in GF–mice at amygdala and prefrontal cortex (Hoban et al., 2017a) and in the striatum (putamen and caudate) (Diaz Heijtz et al., 2011) as well as in post-mortem humans with PD compared to healthy controls (Nair and Ge, 2016).

There is also evidence of the microbiome influence on the host's epigenomics, which is known to influence gene expression, in the context of patho-epigenomics (Bierne, 2017), infection (Hamon and Cossart, 2008; Eskandarian et al., 2013), depression (Tsankova et al., 2006) and drug addiction (Renthal et al., 2007). We have found that *Paenisporosarcina* could influence the epigenetics of the putamen by modifying its histones (GO:0016570) (see Figure S3 and Table S4). Histone deacetylase activity in mice has been observed during stress and depression in the hippocampus (Tsankova et al., 2006) and nucleus accumbens (Renthal et al., 2007). There is growing evidence of microbiota influencing epigenetic changes outside brain tissue (Bierne, 2017) and some mechanisms have been described (Hamon and Cossart, 2008; Eskandarian et al., 2013). Recent evidence has shown dysbiosis associated with epigenetic alterations in cognitive conditions and diseases like autism (Loke et al., 2015), PD (Coppedè, 2012), and many others (Alam et al., 2017).

Eighty two taxa (including the 10 psychobiotics) presented mentions in the cerebral cortex and putamen through the RNA modification/editing ontology (GO:0009451, see Figure S3 and Table S4). It has been found that an epitranscriptomic modification, *N*^6^-methyladenosine (m6A), is highly enriched in miRNAs targets in the mouse brain, and it has an important role in neurodevelopment (Wahlstedt et al., 2009; Meyer et al., 2012). RNA editing has been found to be a key regulator of ion channels in the mouse (Seeburg et al., 2001). As discussed above, these regions could have implications for movement disorders. However, we have not found relevant literature directly associating the MGBa to epitranscriptomics.

Within the *DNA and RNA metabolism* slim, we have found three GOs related to mRNA catabolism (GO:0006402, GO:0000956, and GO:0000184) that associates *Methanobrevibacter* with the cerebellar cortex and the putamen (see Table S4). One of these GOs, labeled “nuclear-transcribed mRNA catabolic process, non-sense-mediated decay” refers to the degradation of mRNAs with a premature stop codon, a process that prevents the translation of potentially harmful proteins (Hentze and Kulozik, 1999). This result suggests a novel microbiota-mediated mechanism of mRNAs cleavage, affecting the expression levels in the brain.

### 3.4. Gut microbiota influencing brain immune system

Strong and consistent evidence has emerged on the association between the host's immune system and the microbiota, which is given by inflammatory mediators. Persistent states of inflammation are also associated with several neurological conditions like depression and anxiety. Evidence shows that inflammatory responses during pregnancy increase the risk of neurodevelopmental conditions like autism spectrum disorders and schizophrenia (Rook et al., 2014).

*Dermabacter* and *Methanobrevibacter* resulted mentioned with the cerebellar cortex by the *Antigen processing* slim (see Table S4). Within this slim, we can find two ontologies associated with the process in which the Major Histocompatibility Complex class I (MHC-I) interacts with a peptide antigen presented in its cell wall (GO:0002474) by the Transporter associated with antigen processing (TAP) pathway (GO:0002479) (see Table S5 and Figure S3). This pathway mediates the translocation of cytosolic peptides into the endoplasmic reticulum that bind to the MHC-I.

Consistent with our results, neuronal expression of MHC-I has been reported in the cerebellum (Letellier et al., 2008; Shatz, 2009). Evidence shows that MHC-I could limit motor learning in the cerebellum, have implications in long-term depression (McConnell et al., 2009) and be associated with the visual system's development and maintenance in marmoset monkeys (Ribic et al., 2011). The expression of this complex is involved in the synaptic plasticity regulation during neurodevelopment (Goddard et al., 2007) and axonal regeneration following injury (Wu et al., 2011). Also, there is evidence of its involvement in neuronal diseases (Pereira and Simmons, 1999; Friese and Fugger, 2005; Chevalier et al., 2011; Kim et al., 2013; Prabowo et al., 2013; Cebrian et al., 2014). A study performed by Mulder et al. showed that low microbiota (hygienic) environment could increase gut expression of MHC-I and other chemokines compared to “natural” environmental acquired microbiota in piglets (Mulder et al., 2009). Our study implicates the microbiota diversity with the expression of MHC-I.

### 3.5. Parkinson's disease

We have found multiple associations with PD (and other motor disorders) through ion channel deficiencies (Mourre et al., 2017; Roeper, 2017), miRNAs (Tan et al., 2013; Nair and Ge, 2016), epigenetic alterations (Coppedè, 2012) and alterations in MHC-I (Cebrian et al., 2014); some of them associating the same cerebral structures like the ones we have found. Our results are particularly interesting given that some of the latter hypothesis of PD etiology has previously involved the microbiota as a relevant and mechanistic factor (Parashar and Udayabanu, 2017).

Gut microbiota have been found altered in subjects with PD, and evidence strongly suggests that it could cause PD through different mechanisms. Reduced organisms found in fecal samples of subjects with PD are *Blautia, Coprococcus*, and *Roseburia* (Keshavarzian et al., 2015) and the psychobiotic *Prevotella* (Scheperjans et al., 2015). Hill-Burns et al., recently reported altered abundances of the psychobiotics *Bifidobacterium, Lactobacillus* and *Faecalibacterium*, and non-psychobiotics *Blautia, Roseburia* and *Akkermansia* genus (Hill-Burns et al., 2017). Another recent study found decreased *Blautia, Faecalibacterium* and *Ruminococcus*, and increased *Escherichia-Shigella, Streptococcus, Proteus*, and *Enterococcus* as in comparison with controls (Li et al., 2017).

In this context, by considering the most abundant GOs for each taxa, nine bacterial genera (*Lactobacillus, Bifidobacterium, Coprococcus, Prevotella, Ruminococcus, Escherichia, Streptococcus, Proteus*, and *Enterococcus*) are associated with potassium ion channels; three of them (*Faecalibacterium, Blautia, Roseburia*) are related to translational termination and RNA modification, and two (*Ruminococcus, Roseburia*) are also associated with axonogenesis. However, other functional associations could be found at the Table S4.

The *Methanobrevibacter* also have been found to influence the spliceosome at PD-associated brain substructures. We have not found any associations of this taxon with PD, however, most of the microbiota profiling projects are 16S-rRNA-based, and they missed archaea organisms.

Despite the extensive literature on PD and that we have found many coincidences for this disease, the results presented here could pave the way for novel hypotheses on PD pathophysiology.

## 4. Conclusions

In this work, we have presented an *in silico* framework to associate metaproteins with brain expression data through ontological labels. Also we have defined *a posteriori* GO slims based on semantic similarity clustering. This data-driven study suggests that microbiota could affect synapse and voltage-gated ion channels in brain structures, which have been related to movement disorders, like the basal ganglia. Beacuse of the GO associations, we can suggest that microbiota have an influence on DNA and RNA metabolism. Given the strong association of *Methanobrevibacter* with spliceosome GOs, we suggest that mechanisms involving miRNAs and mRNA catabolism may have a role in several brain structures. This last taxon along with *Dermabacter* were found associated with the MHC-I through the TAP pathway in the cerebellar cortex. We also found associations like *Paenisporosarcina* with histone modification, and with many other taxa, including known psychobiotics, as RNA modificators. Parkinson's disease was coincidently found associated to several taxa, brain structures, and functional slims related with neuronal communication, DNA/RNA metabolism and alterations in the MHC-I.

This work is a novel systems approach based on T2BS functional annotations, where we used large, specialized databases to discover possible mechanisms where the microbiota could influence specific brain regions. Our results could also inspire germ-manipulation studies to find therapeutic approaches on neurological movement disorders.

## 5. Materials and methods

### 5.1. Data curation

**Figure 5:**
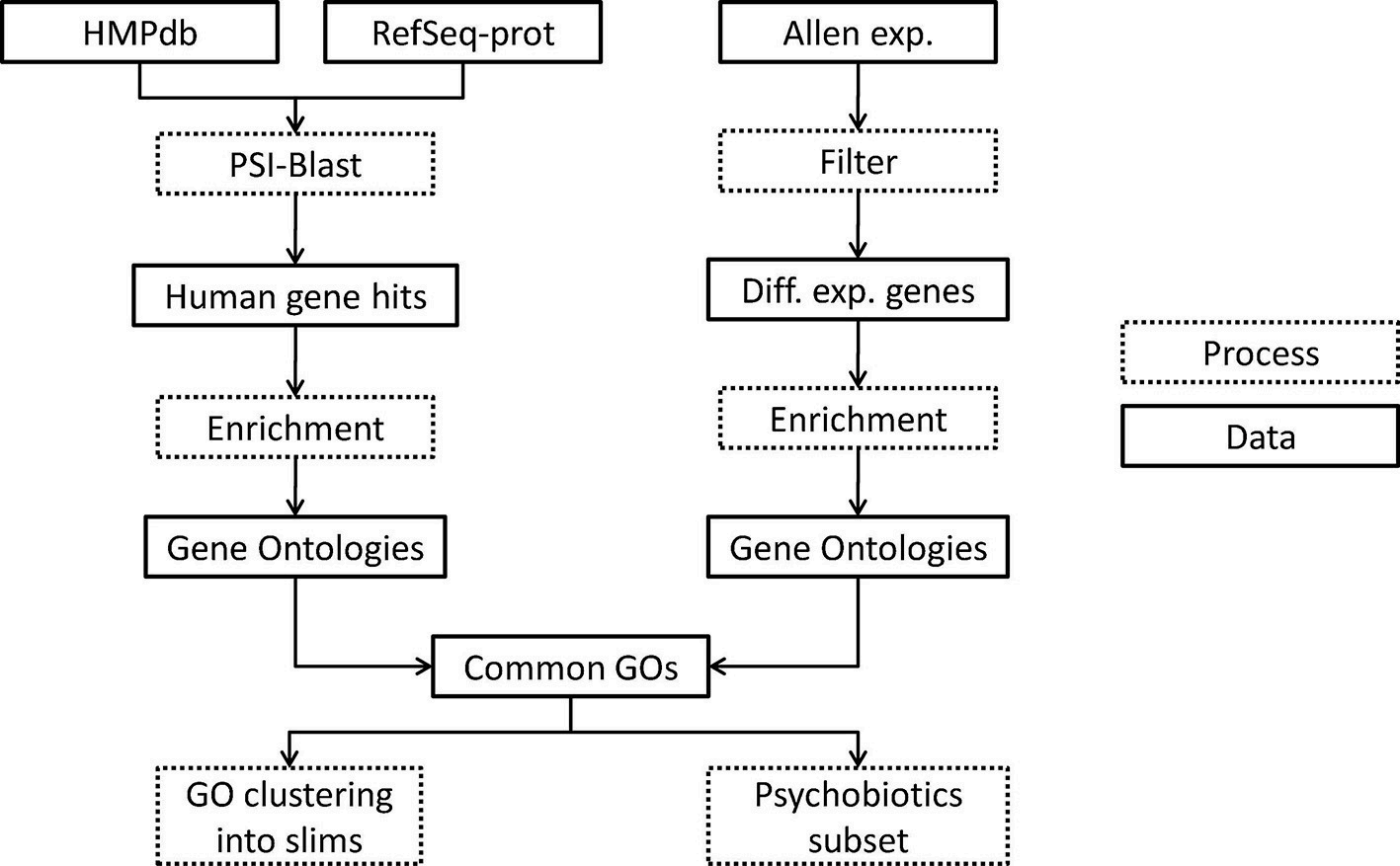
Flowchart of the methodology used. Dotted boxes indicate processing steps and regular boxes are data downloaded or resulted from a process. HMPdb, Human Microbiome Project database; PSI, Position Specific Iterative; GO, Gene Ontology.

Gastrointestinal tract microbiota proteome (metaproteome) of database (Reference Genome sequence data obtained from 300 subjects) was downloaded from the Human Microbiome Project website^1^ as contigs (see Figure 5, database “HMPdb”). The human protein reference sequences (RefSeq-prot) database was downloaded from the NCBI ftp server^2^ (see Figure 5, data “RefSeq-prot”). Also, post-mortem human brain RNA-sequencing dataset (donor H0351.2001) was downloaded from the Allen Brain Atlas web page^3^ (see Figure 5, data “Allen exp.”), which contains three or four replicates per brain substructure.

The metaproteome files were merged at the genus level to generate a single non-redundant file per taxon. These files were used as query for the Position Specific Iterative (PSI)-Blast local and the RefSeq-prot was used as database (see Figure 5, process “PSI-Blast”). PSI-Blast is an iterative version of protein blast to find highly conservative relationships between proteins. PSI-Blast parameters were set up to 10 iterations (maximum) and *e*-value threshold ≤ 0.05. The PSI-Blast results by taxa were obtained in one file each (see Figure 5, data “Human gene hits”). The human protein hits of the last iteration were extracted from the files and redundancies removed. Each list of non-redundant proteins was annotated with its geneID by using the GCRh38 database.

The human RNA-seq database at the Allen Brain Atlas contains normalized expression data on 22,318 genes. To see the normalization methods used go to documentation at brain-map.org. Genes not annotated in Entrez database or with zero counts in all samples were eliminated. Genes with CPM ≤ 0.5 in at least two replicates of the same brain sub-structure were also eliminated. We calculated the Euclidian distances between samples by using a multidimensional scaling with the function plotMDS of the edgeR library, scaling with the top 500 genes with larger log2-fold changes. Afterwards, we selected those genes within each substructure with differential expression compared to the mean across all samples by using the methods explained in Lun and Smyth (2015) using the edgeR library (McCarthy et al., 2012). For the latter step we first estimated the biological and technical variability of the reads by using the glmQLFit function, which performs a gene-wise negative binomial generalized linear model with quasi-likelihood method (Lun and Smyth, 2015). Afterwards, we used a quasi-likelihood *F*-test (substructure CPMs vs. the mean CPMs) due to its rigid error rate control at including the uncertainty in the estimation of the dispersion. The multiple comparisons problem (which states that when many hypothesis are tested, the chance of erroneous conclusions increases) was corrected by Bonferroni method, and only the genes with *p* ≤ 0.05 were preserved. Also, only genes with absolute log-fold change ≥1.5 were preserved (see Figure 5, process “Filter” and Data “Diff. exp. genes”).

### 5.2. Gene ontology enrichment and common ontology

Each gene list associated to taxa or brain substructure was enriched using python's goatools^4^ find_enrichment.py function to find the GOs statistically associated to the list of genes (α = 0.05) (see Figure 5, process “Enrichment”). Ontologies with Bonferroni corrected *p* ≤ 0.05 were selected. Statistically significant underrepresented GOs were discarded in the taxon associated gene lists. This resulted in a set of ontologies associated to each taxon and each brain sub-structure (see Figure 5, data “Gene Ontologies”).

We annotated the T2BS common ontologies. This resulted in a T2BS association list of GOs with annotated genes (see Figure 5, data “Common GOs”).

### 5.3. Analysis

For each pair of T2BS we calculated the Sorensen-Dice coefficient (similarity measure between two samples) and applied hierarchical clustering to observe the distribution of the common GOs. Also we applied Pearson's correlation (coefficient of linear correlation) to the number of genes found in each taxon to the number of common GO terms found in the same taxon.

From all of the GOs obtained, we calculated its semantic similarity by the goatools function semantic_similarity.py. This measure is defined as the reciprocal of the minimal number of branches (or edges) between two GO terms in the GO topology. It can also be defined as the reciprocal of the shortest path between two GO terms by using graph theory argot. We grouped GO terms with similar functions by manually curating clusters obtained by hierarchical clustering the semantic similarities between all GOs; to refer to these groups we use “slims” (see Figure 5, process “GO clustering into slims”). From the set of taxa we selected those known as psychobiotics according to literature to perform a deeper exploratory data analysis (see Figure 5, process “Psychobiotics subset”).

## Author contributions

AF and JM developed the main idea of the work, but the pipeline was finally designed by the three authors. The three authors participated in the figure design, discussion, and the final draft. AF performed the experiments, analyzed the results and wrote the initial draft of introduction, methods, and results. He also participated in the section 3 with an emphasis on the slims and Parkinson's disease. AM also participated in the discussion with an emphasis on the microbiota and psychobiotics. JM also participated in the discussion with a systems approach and edited the figures.

### Conflict of interest statement

The authors declare that the research was conducted in the absence of any commercial or financial relationships that could be construed as a potential conflict of interest.

## Footnotes

^1^https://www.hmpdacc.org/hmp/

^2^https://ftp.ncbi.nlm.nih.gov/refseq/H_sapiens/mRNA_Prot/

^3^http://human.brain-map.org/static/download

^4^https://github.com/tanghaibao/goatools
