# Supplementary material for: An Ontology Systems Approach on Human Brain Expression and Metaproteomics
Source: Front Microbiol. 2018 Mar 8;9:406. doi: 10.3389/fmicb.2018.00406 (PMC5852110; doi:10.3389/fmicb.2018.00406)
Supplement: Supplementary file 6 [file Image1.pdf]

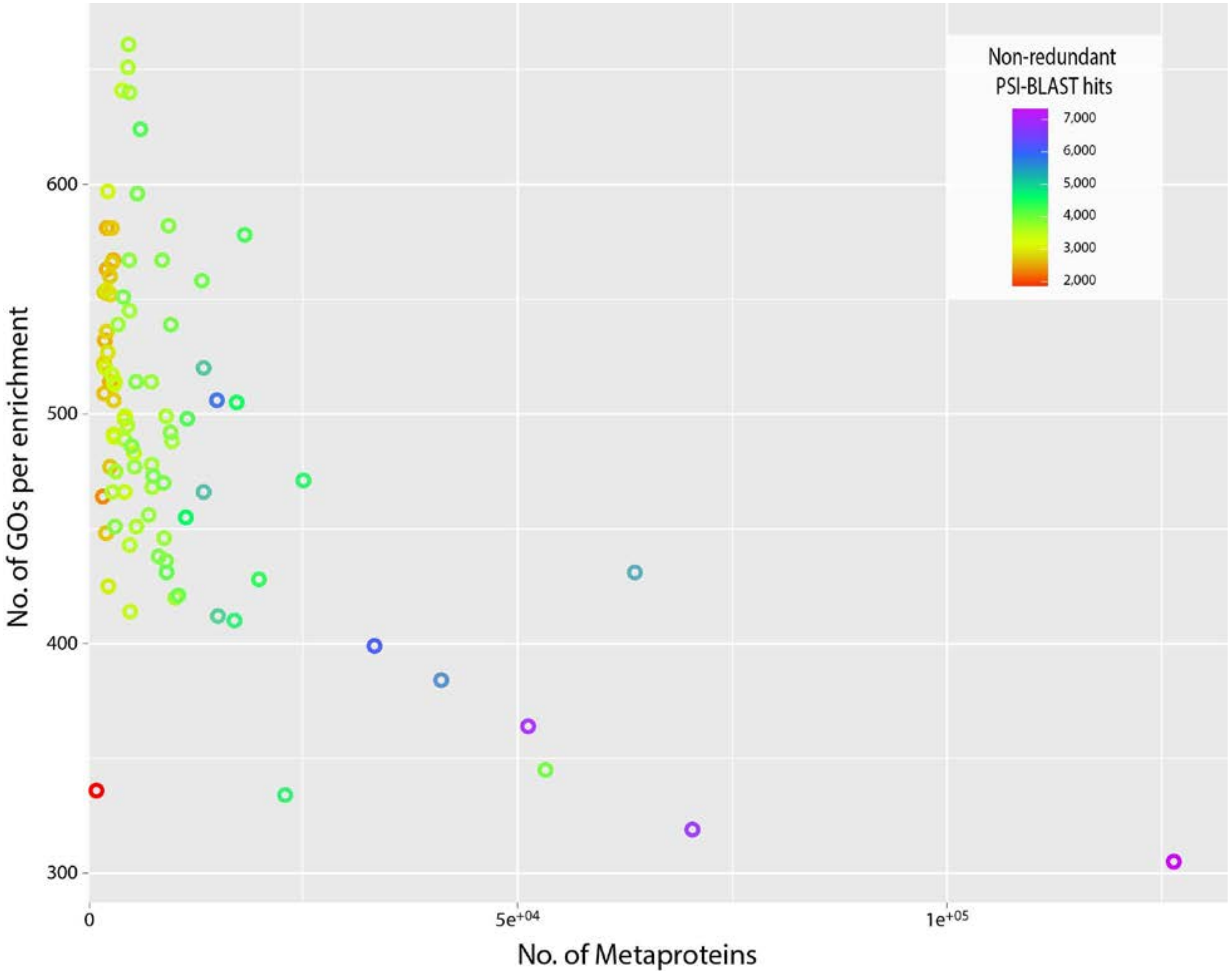

Figure S1. Scatterplot of the number of metaproteins of each taxon (92) with the number of non-redundant human proteins found by the PSI-blast algorithm using the metaproteins. The color-coded information represents the number of Gene ontologies (GOs) found by the enrichment analysis.

Flores-Saiffe, et al. An ontological systems approach on human brain expression and metaproteomics. Frontiers in Microbiology (2017).
