# Supplementary material for: An Ontology Systems Approach on Human Brain Expression and Metaproteomics
Source: Front Microbiol. 2018 Mar 8;9:406. doi: 10.3389/fmicb.2018.00406 (PMC5852110; doi:10.3389/fmicb.2018.00406)
Supplement: Supplementary file 8 [file Image3.pdf]

Slim: *Antigen _processing*

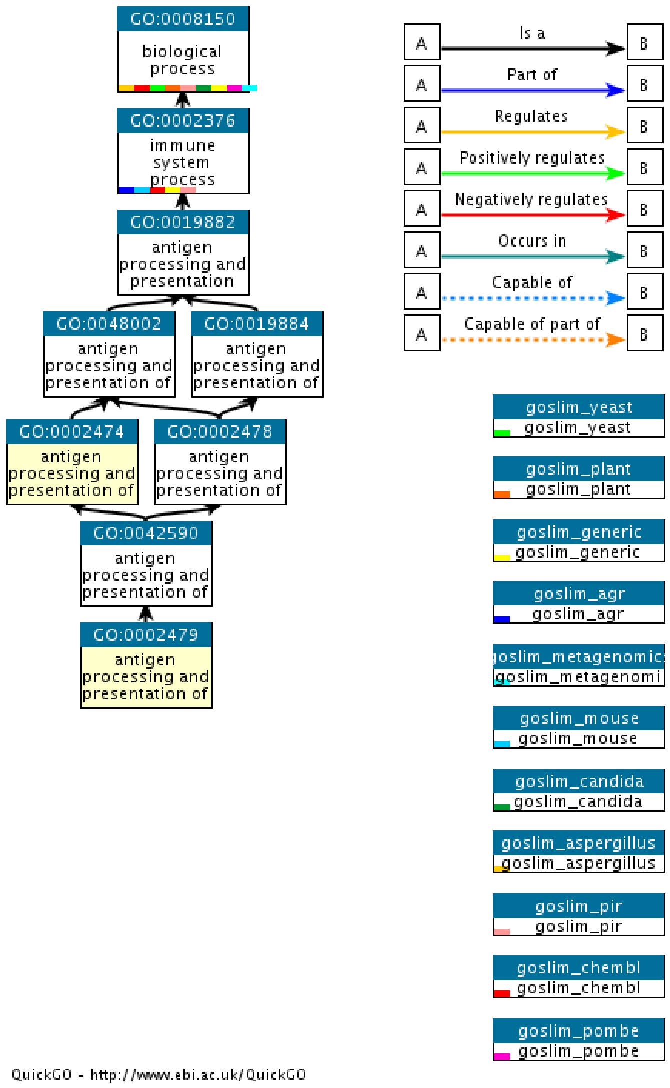

Slim: *Binding*

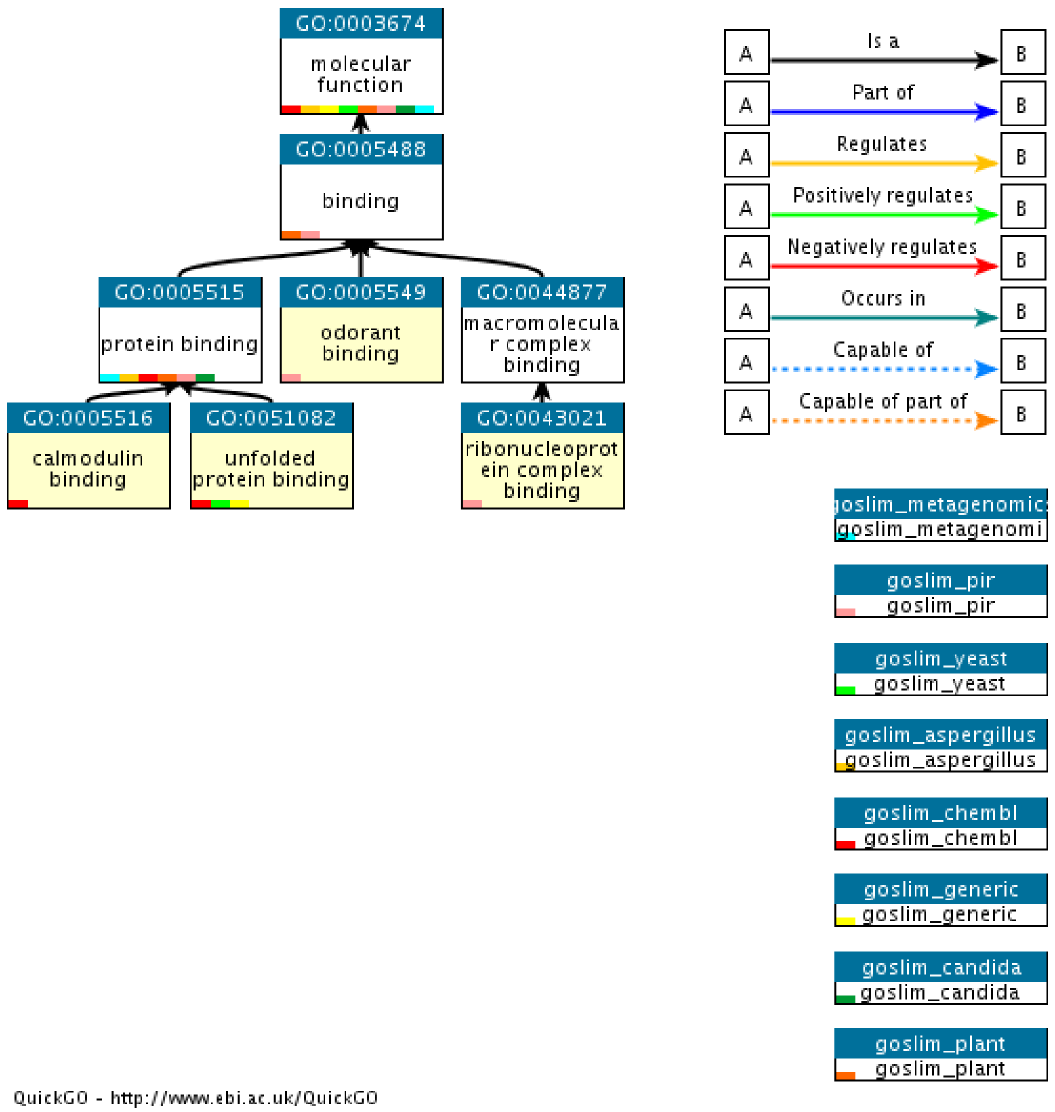

Slim: *Cellular_component_organization*

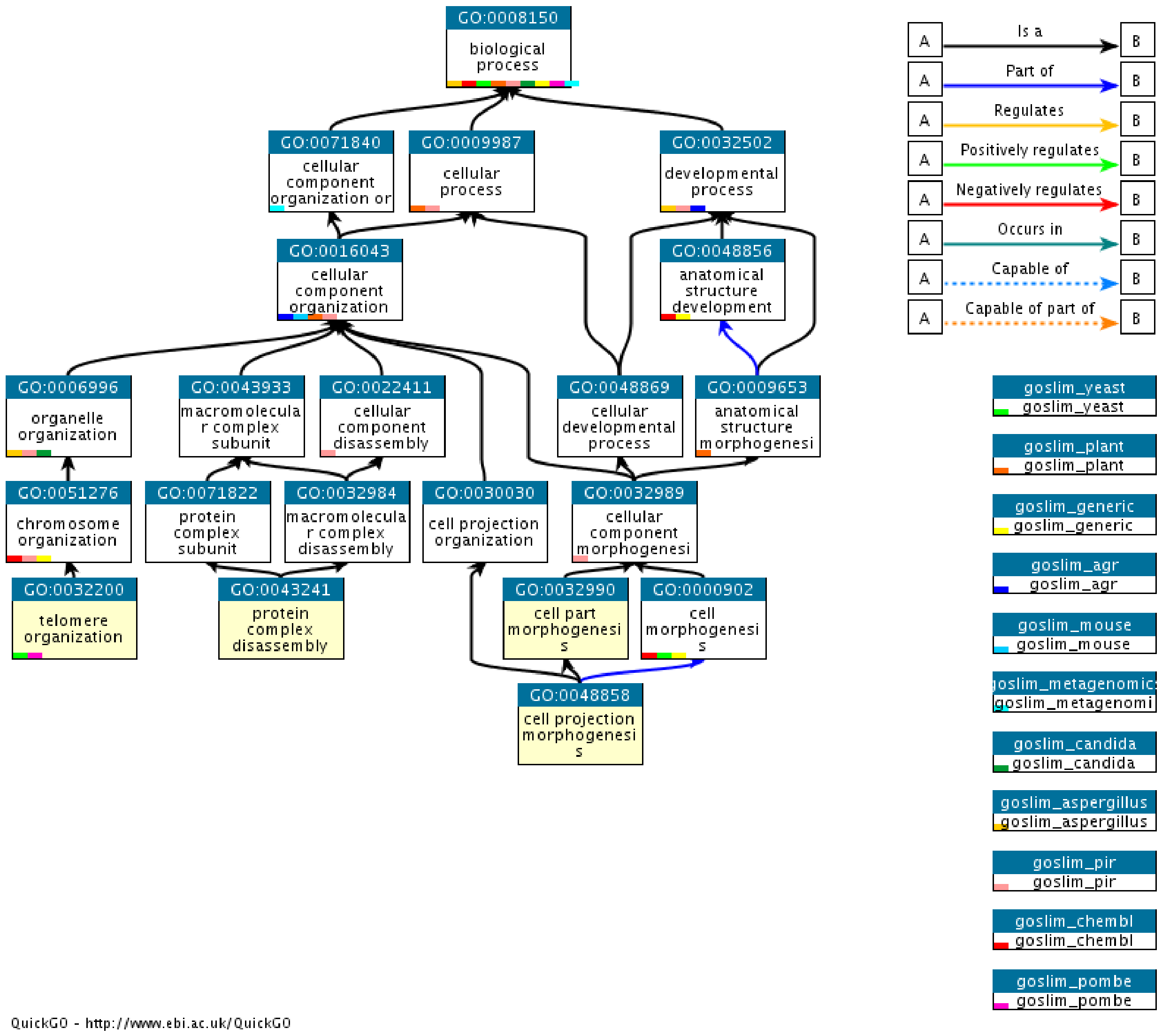

Slim: *DNA_RNA_metabolism*

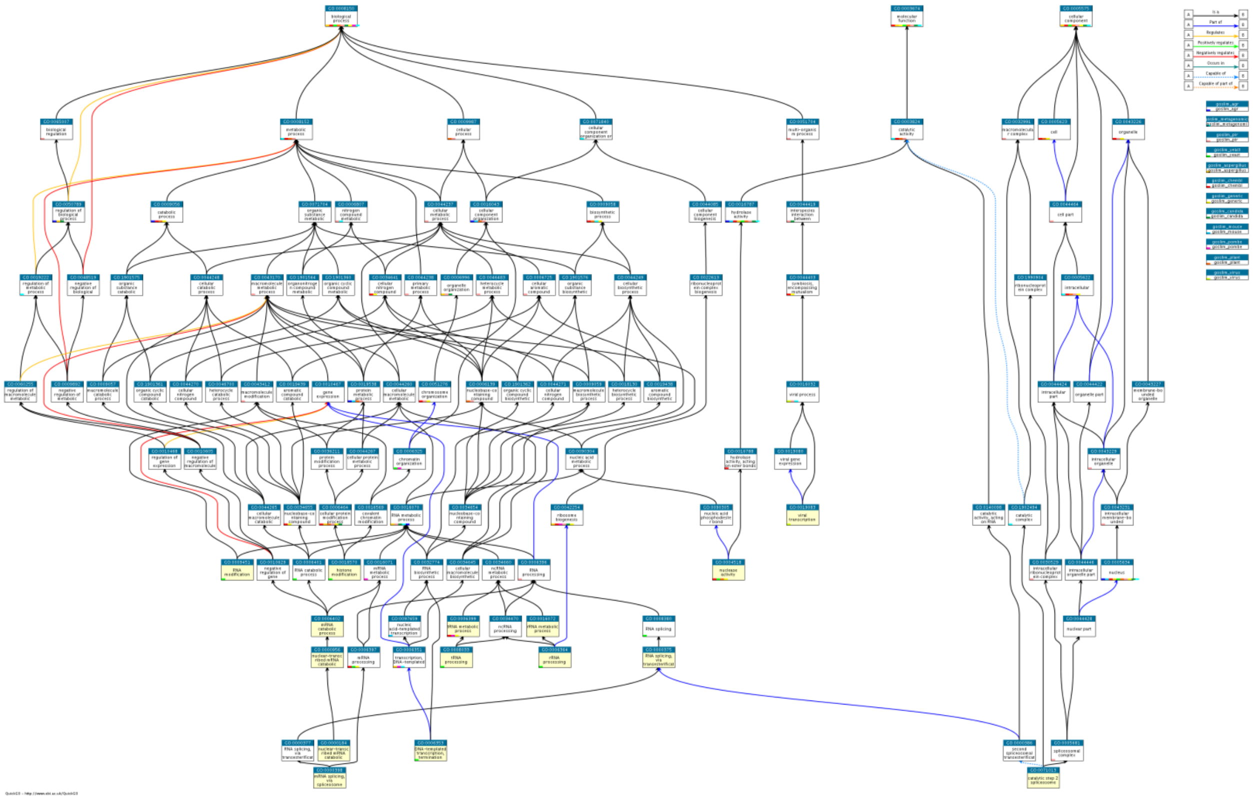

Slim: Intracellular_part

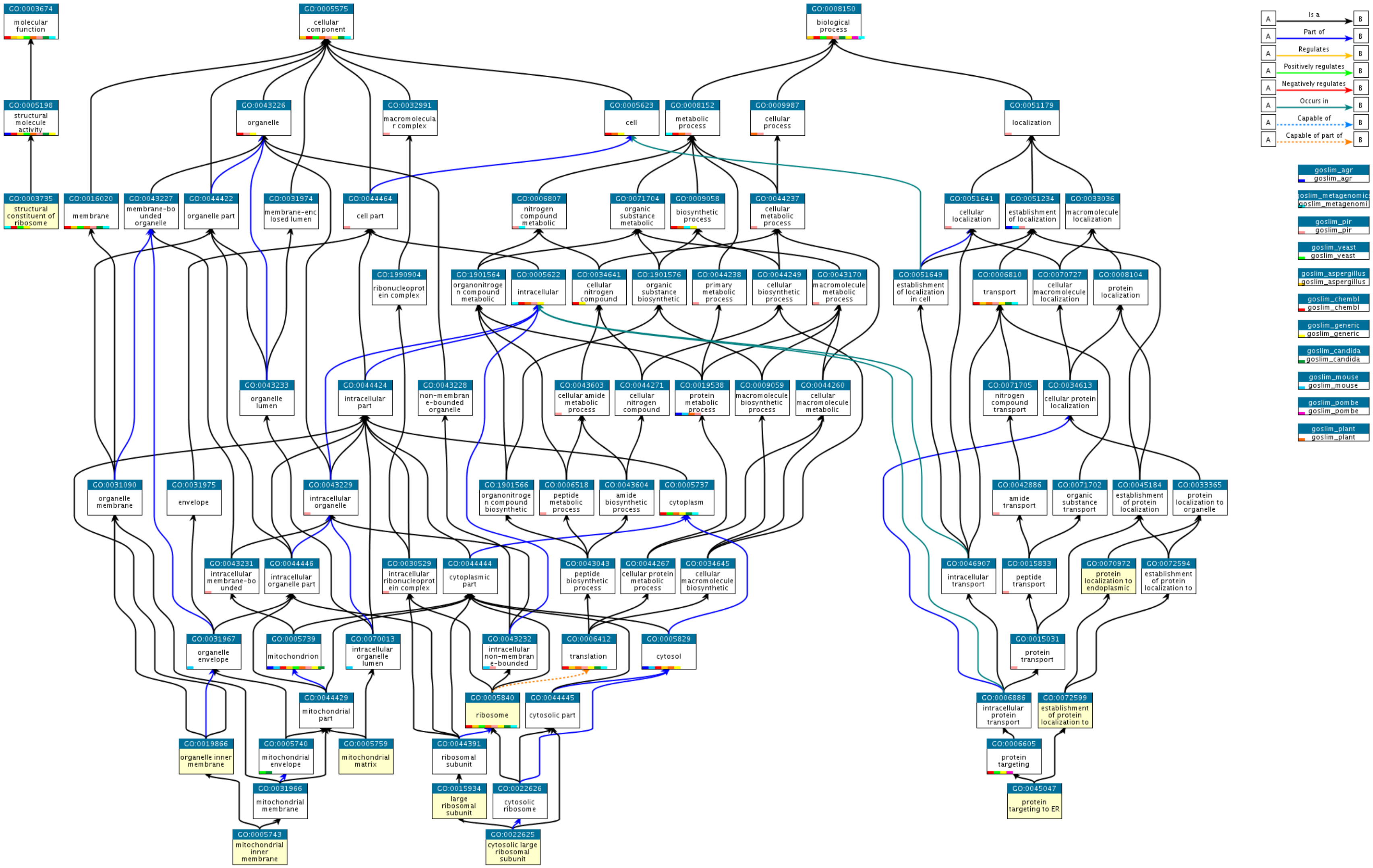

QuickGO - http://www.ebi.ac.uk/QuickGO

Slim: *Membrane_potential*

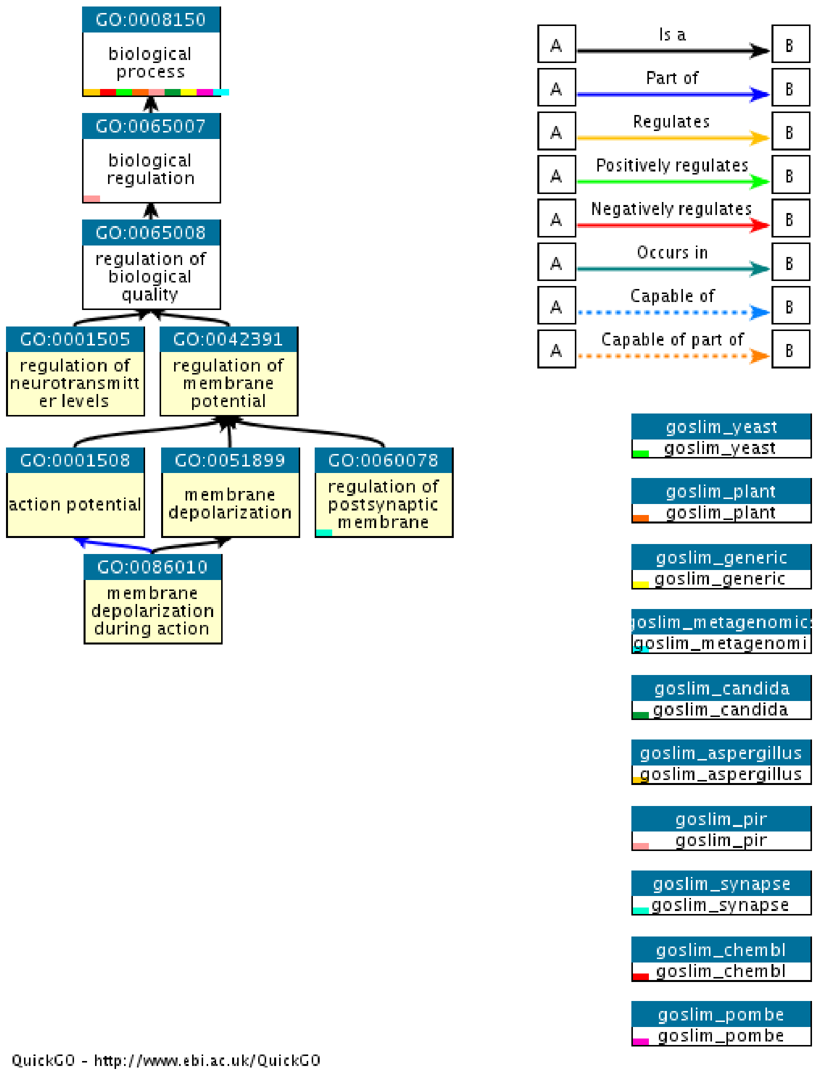

Slim: *Ion_Transport*

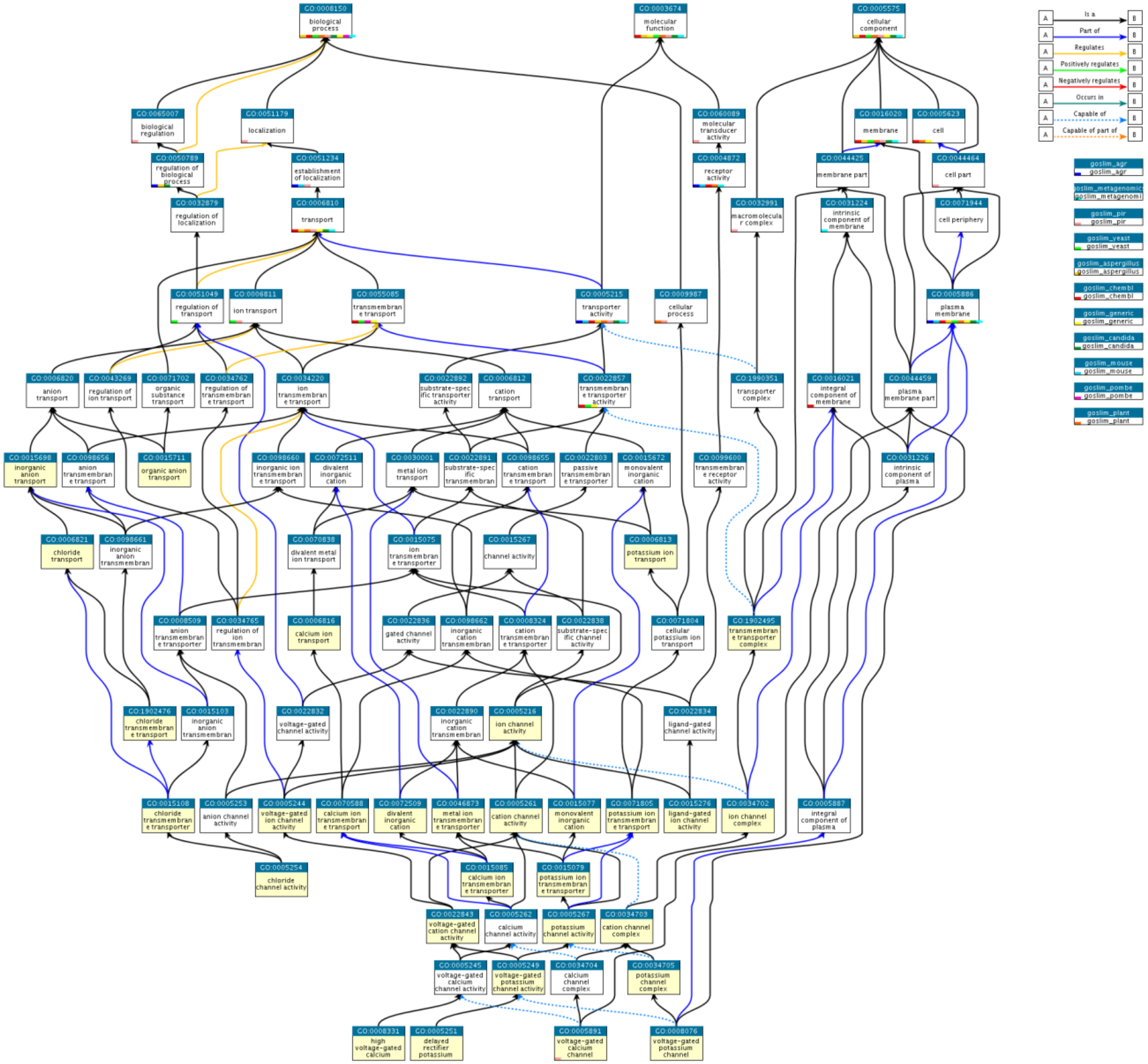

QuickGO - http://www.ebi.ac.uk/QuickGO

Slim: *Others*

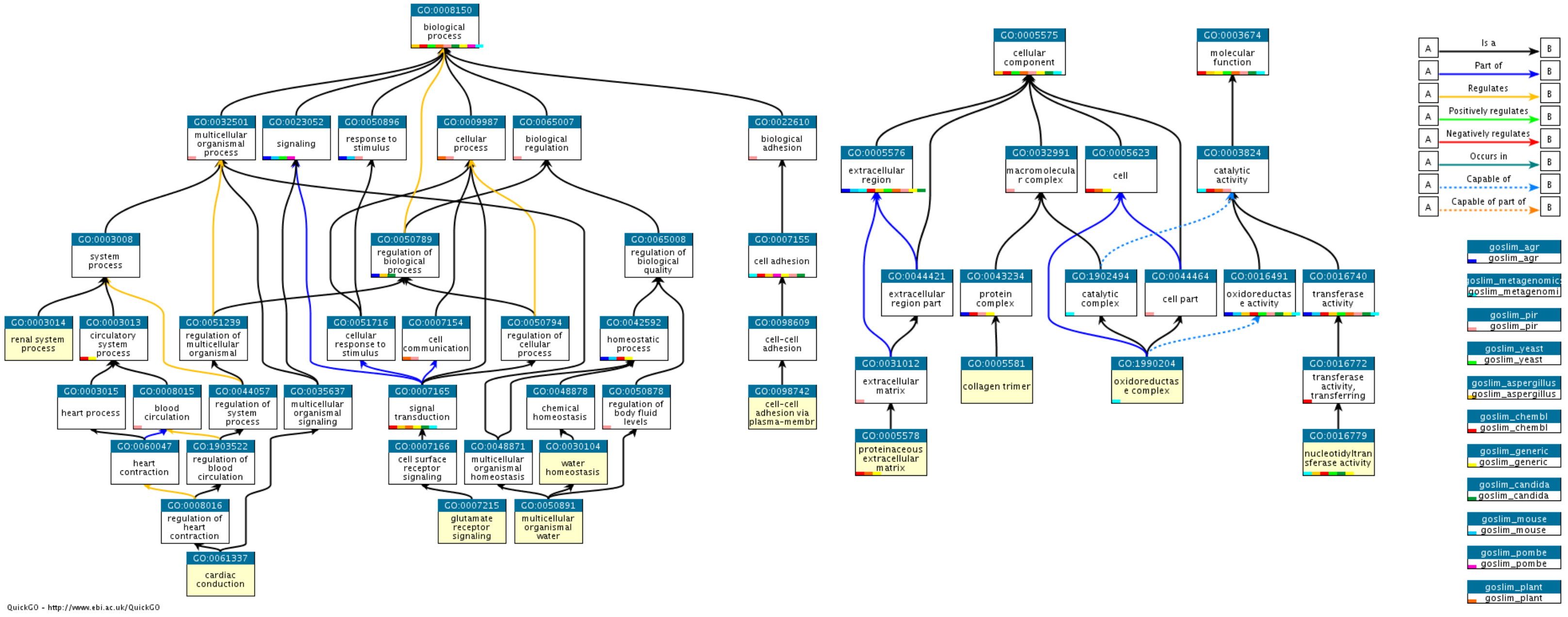

GO:0008150 biological process
GO:0032501 multicellular organismal process
GO:0023052 signaling
GO:0050896 response to stimulus
GO:0009987 cellular process
GO:0065007 biological regulation
GO:0022610 biological adhesion
GO:0003008 system process
GO:0050789 regulation of biological process
GO:0065008 regulation of biological quality
GO:0007155 cell adhesion
GO:0003014 renal system process
GO:0003013 circulatory system process
GO:0051239 regulation of multicellular organismal
GO:0051716 cellular response to stimulus
GO:0007154 cell communication
GO:0050794 regulation of cellular process
GO:0042592 homeostatic process
GO:0098609 cell-cell adhesion
GO:0003015 heart process
GO:0008015 blood circulation
GO:0044057 regulation of system process
GO:0035637 multicellular organismal signaling
GO:0007165 signal transduction
GO:0048878 chemical homeostasis
GO:0050878 regulation of body fluid levels
GO:0098742 cell-cell adhesion via plasma-membr
GO:0060047 heart contraction
GO:1903522 regulation of blood circulation
GO:0007166 cell surface receptor signaling
GO:0048871 multicellular organismal homeostasis
GO:0030104 water homeostasis
GO:0008016 regulation of heart contraction
GO:0007215 glutamate receptor signaling
GO:0050891 multicellular organismal water
GO:0061337 cardiac conduction
QuickGO - http://www.ebi.ac.uk/QuickGO
GO:0005575 cellular component
GO:0003674 molecular function
GO:0005576 extracellular region
GO:0032991 macromolecula r complex
GO:0005623 cell
GO:0003824 catalytic activity
GO:0044421 extracellular region part
GO:0043234 protein complex
GO:1902494 catalytic complex
GO:0044464 cell part
GO:0016491 oxidoreductas e activity
GO:0016740 transferase activity
GO:0031012 extracellular matrix
GO:0005581 collagen trimer
GO:1990204 oxidoreductas e complex
GO:0016772 transferase activity, transferring
GO:0005578 proteinaceous extracellular matrix
GO:0016779 nucleotidyltran sferase activity
A Is a B
A Part of B
A Regulates B
A Positively regulates B
A Negatively regulates B
A Occurs in B
A Capable of B
A Capable of part of B
goslim_agr
goslim_metagenomics
goslim_pir
goslim_yeast
goslim_aspergillus
goslim_chembl
goslim_generic
goslim_candida
goslim_mouse
goslim_pombe
goslim_plant

Slim: *Neurogenesis*

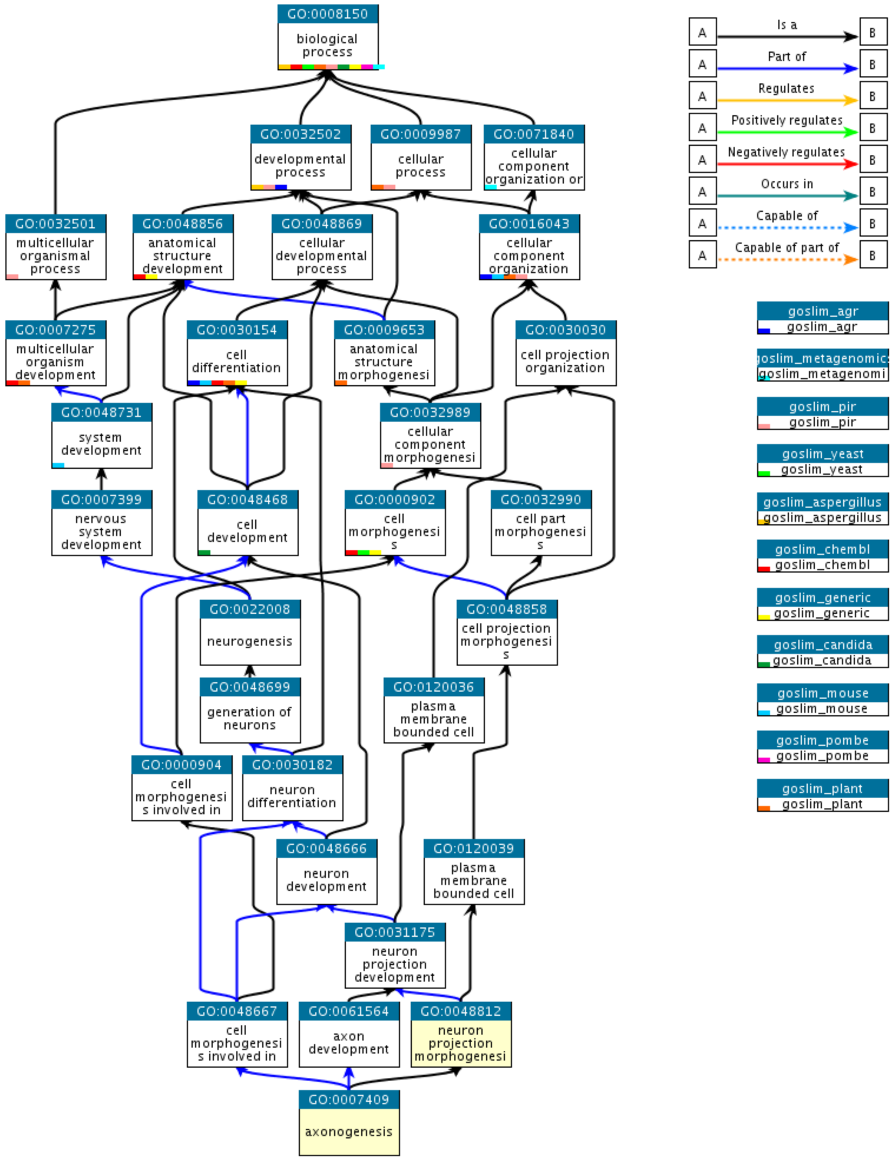

QuickGO - http://www.ebi.ac.uk/QuickGO

Slim: *Protein_membrane_transport*

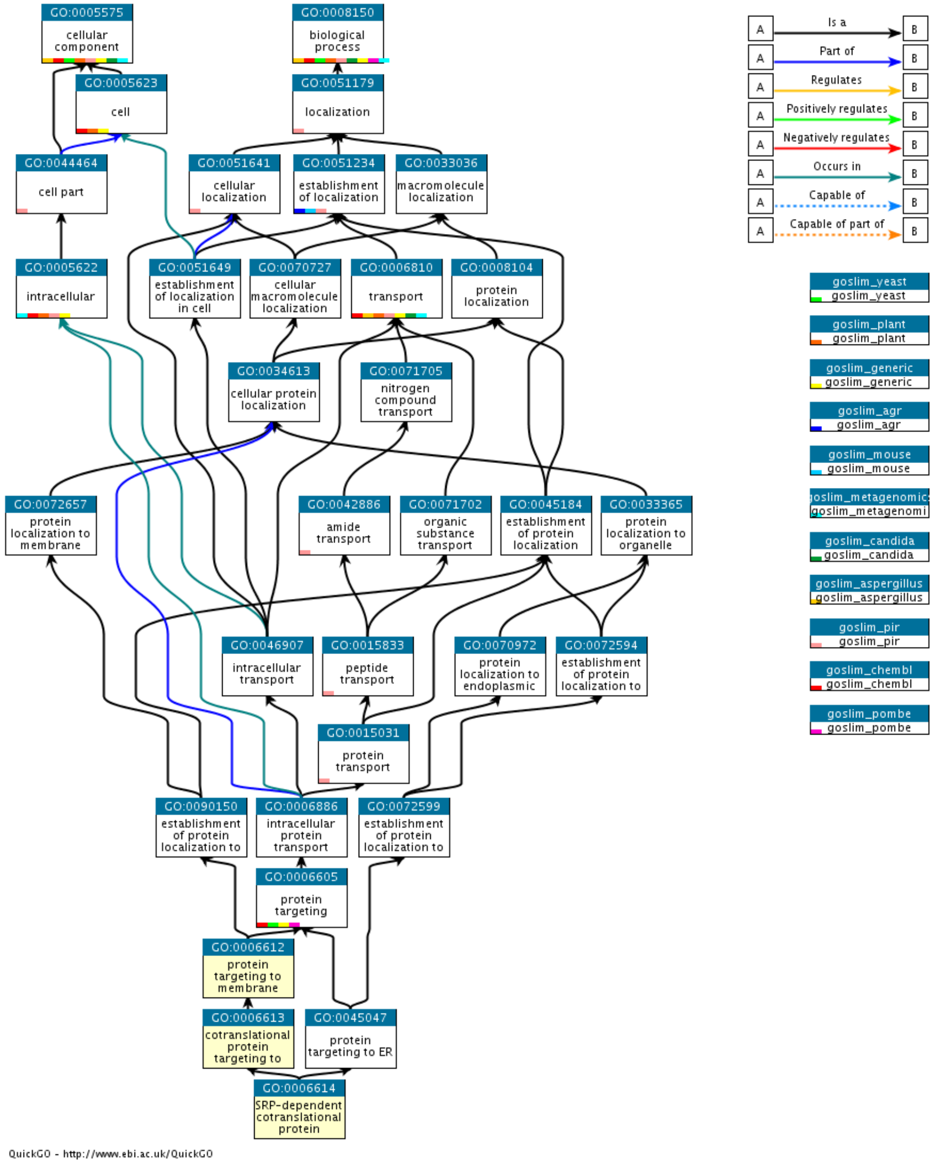

Slim: *Protein_metabolism*

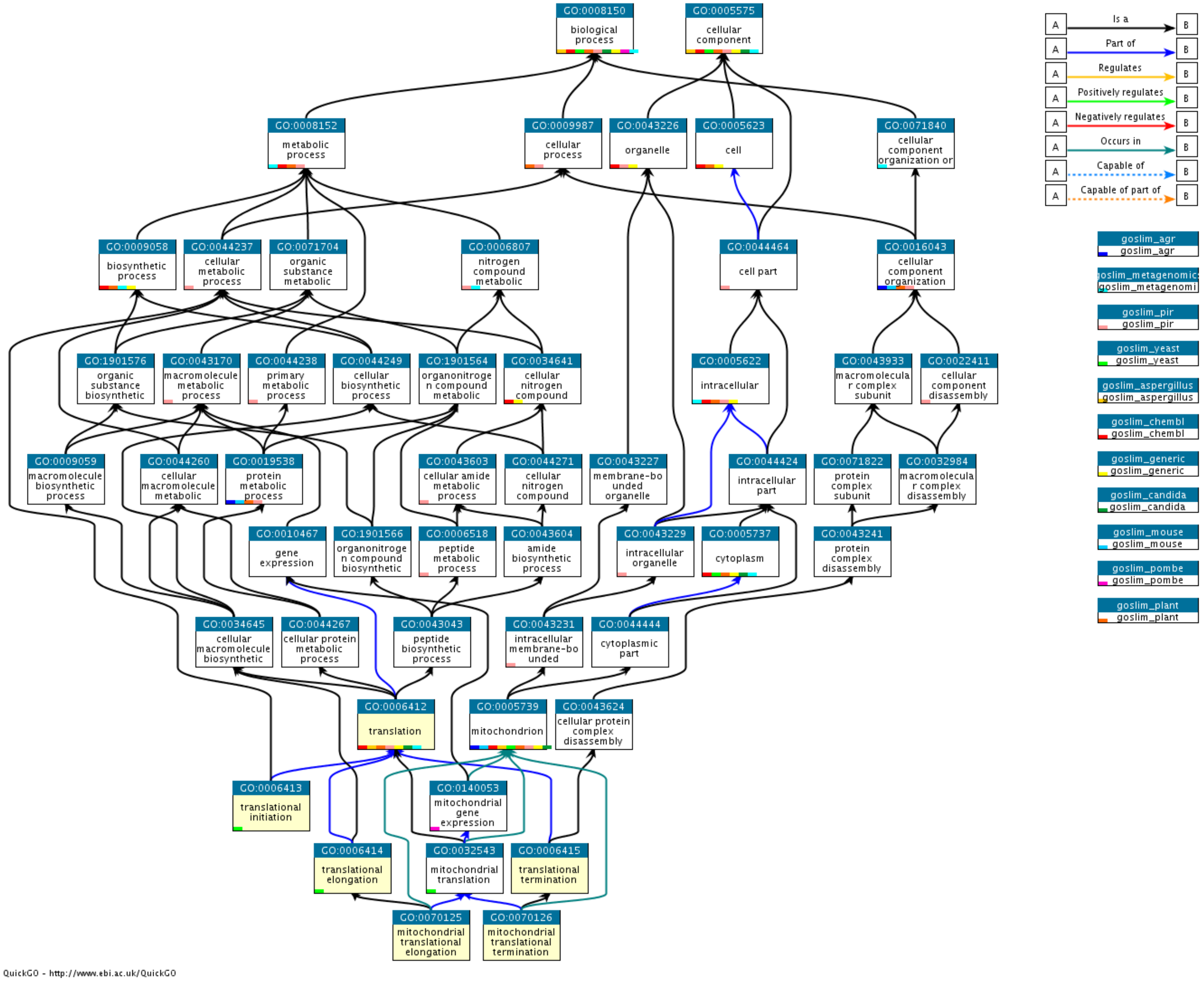

Slim: *Synapse_organization*

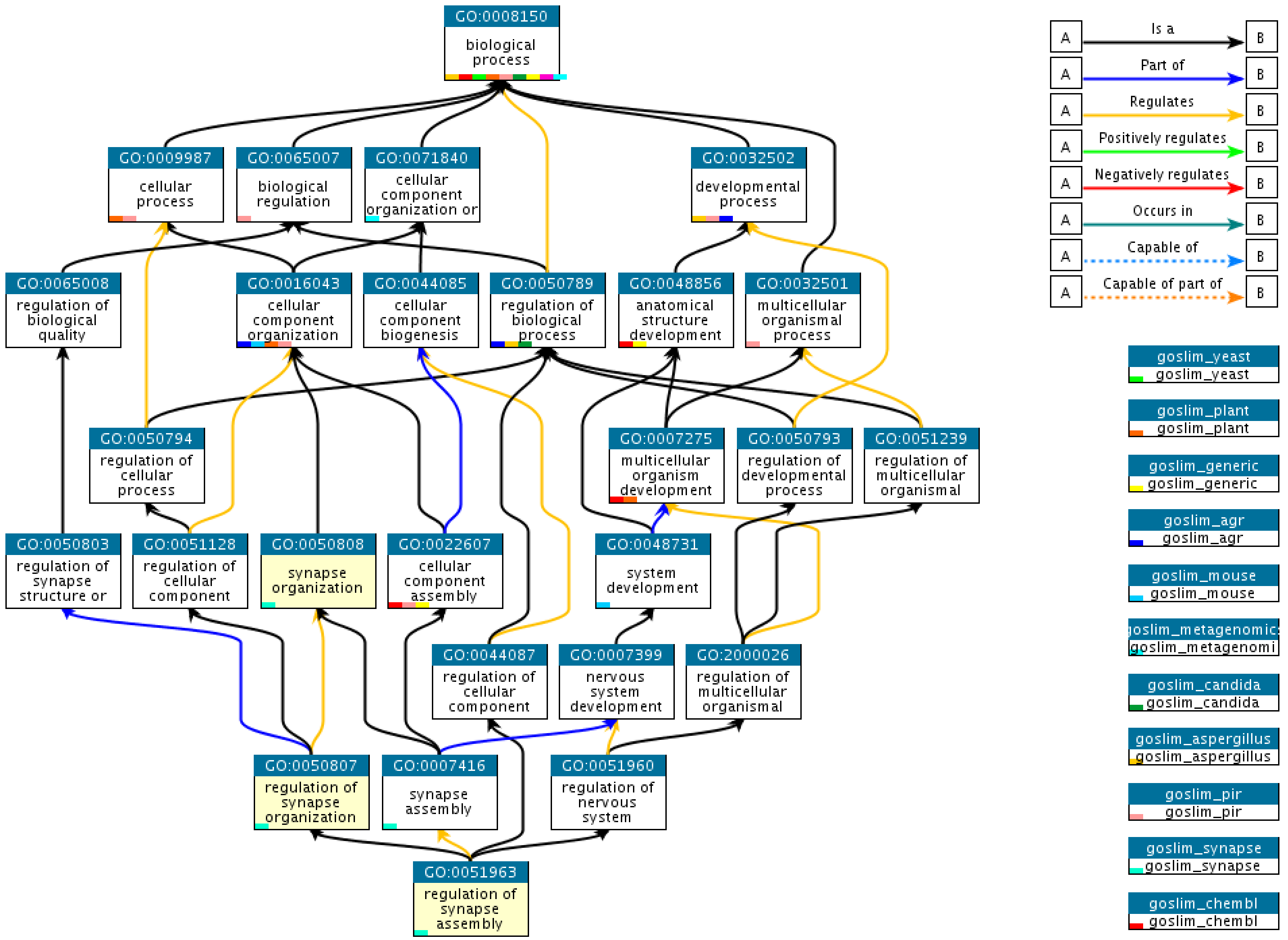

QuickGO - http://www.ebi.ac.uk/QuickGO

Slim: *Synapse*

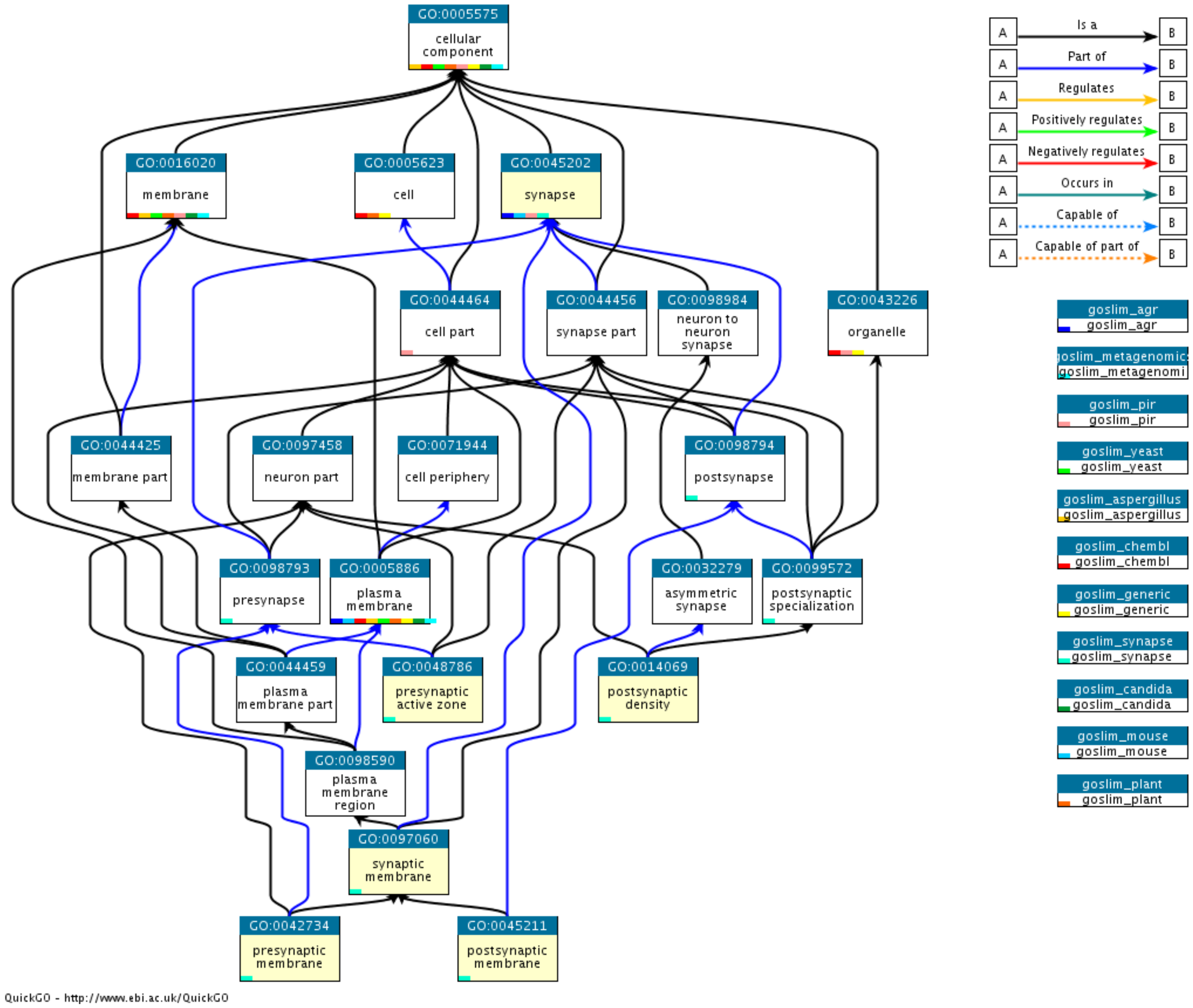

Slim: *Transport_(Others)*

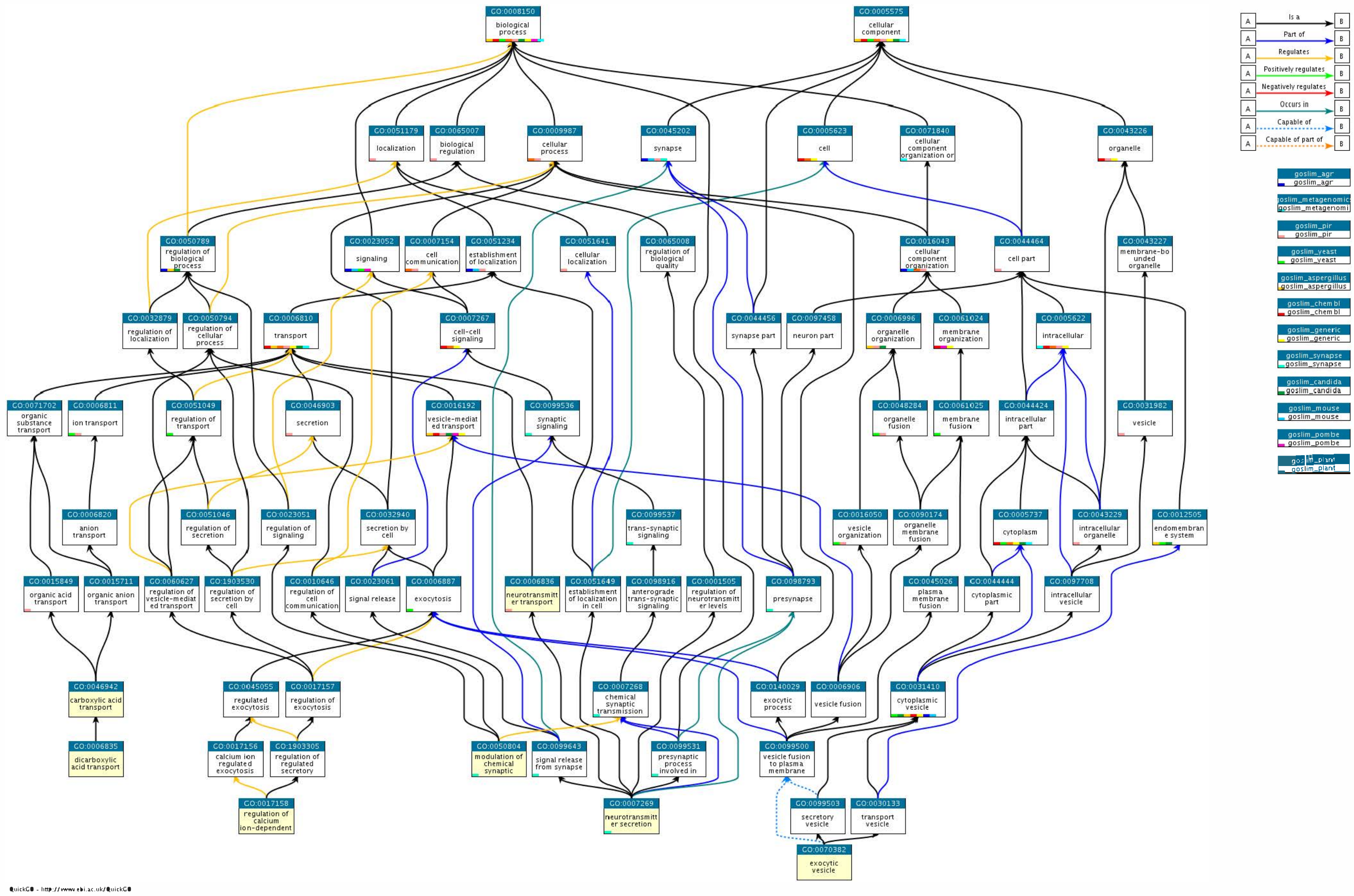

Figure S3. This figure contains the ontological maps of all slims: Antigen processing, Binding, Cellular component organization, DNA and RNA metabolism, Intracellular part, Membrane potential, Ion transport, Other, Neurogenesis, Protein membrane transport, Protein metabolism, Synapse organization, Synapse, and Transport (others).
