# Supplementary material for: An Ontology Systems Approach on Human Brain Expression and Metaproteomics
Source: Front Microbiol. 2018 Mar 8;9:406. doi: 10.3389/fmicb.2018.00406 (PMC5852110; doi:10.3389/fmicb.2018.00406)
Supplement: Supplementary file 7 [file Image2.pdf]

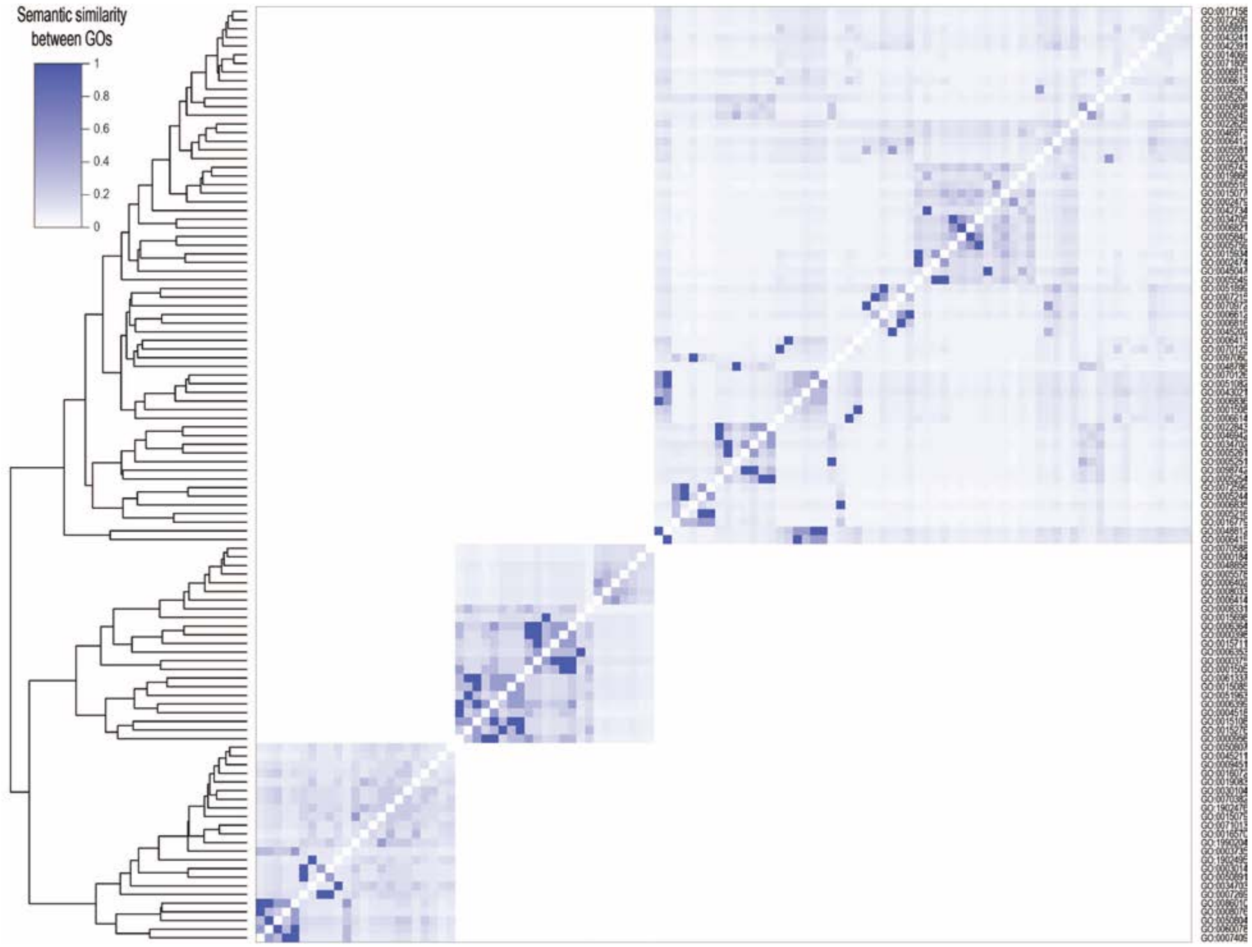

Figure S2. Semantic similarity heatmap hierarchically clustered across all the common taxon-brain substructure Gene ontologies (GOs). It is interesting to note some distinct semantic groups of GOs.

Flores-Saiffe, et al. An ontological systems approach on human brain expression and metaproteomics. Frontiers in Microbiology (2017).
